# Supplementary material for: High-Performance Ultraviolet Photodetector Based on a Zinc Oxide Nanoparticle@Single-Walled Carbon Nanotube Heterojunction Hybrid Film
Source: Nanomaterials (Basel). 2020 Feb 24;10(2):395. doi: 10.3390/nano10020395 (PMC7075298; doi:10.3390/nano10020395)
Supplement: Supplementary file 1 [file nanomaterials-10-00395-s001.pdf]

Supplementary Materials

High-performance ultraviolet photodetector based on a zinc oxide nanoparticle@single-walled carbon nanotube heterojunction hybrid film

Myung-Soo Choi[1], Taehyun Park[1], Woo-Jae Kim[2*], and Jaehyun Hur[1*]

[1]Department of Chemical and Biological Engineering, Gachon University, Seongnam, Gyeonggi 13120, Republic of Korea

[2]Department of Chemical Engineering and Materials Science, Ewha Womans University, Seoul 03760, Korea

---

*Corresponding authors. Prof. W.-J. Kim (Tel: +82-2-3277-4327; Fax: +82-2-3277-4213) and Prof. J. Hur (Tel: +82-31-750-5593; Fax: +82-31-750-8839)

E-mail addresses: wjkim1974@ewha.ac.kr and jhhur@gachon.ac.kr

(a) ZnO@A-SWNT (ratio of 40:1)

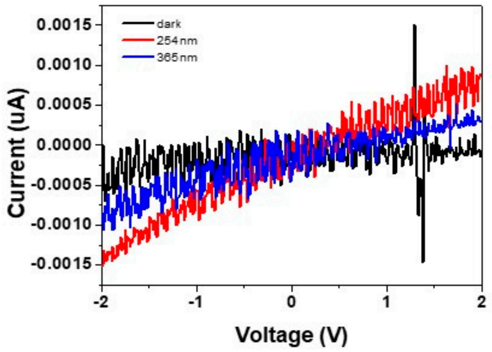

(b) ZnO@A-SWNT (ratio of 28:1)

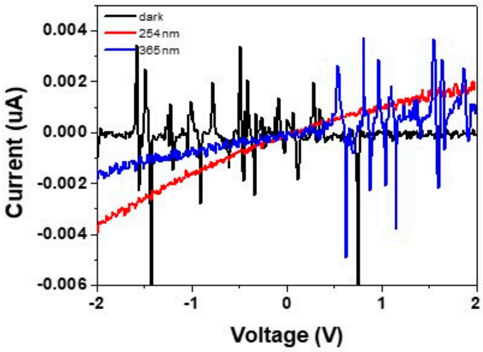

(c) ZnO@H-SWNT (ratio of 40:1)

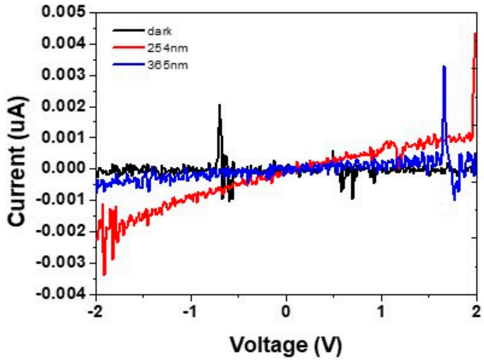

(d) ZnO@H-SWNT (ratio of 28:1)

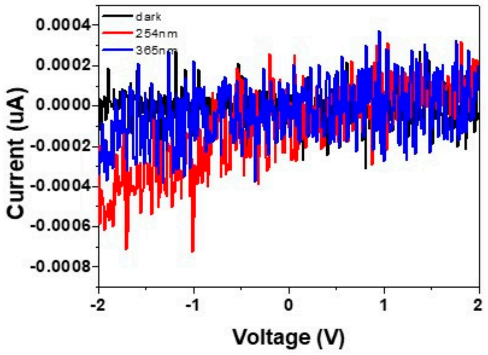

(e) ZnO@C-SWNT (ratio of 40:1)

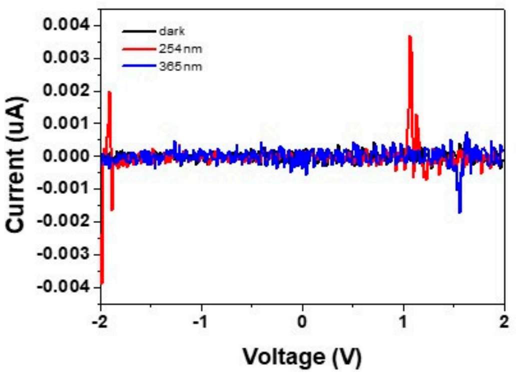

(f) ZnO@C-SWNT (ratio of 28:1)

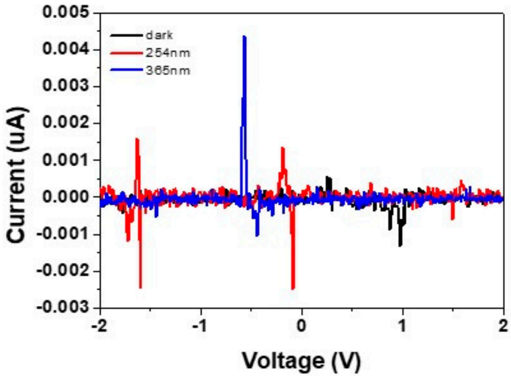

Fig. S1. I-V measurements for ZnO@SWNT with A-SWNTs, H-SWNTs, and C-SWNTs at the ratios of 40:1 and 28:1 (ZnO/SWNT, w/w) under 254 and 365 nm UV irradiation (0.47 mW/cm^2).

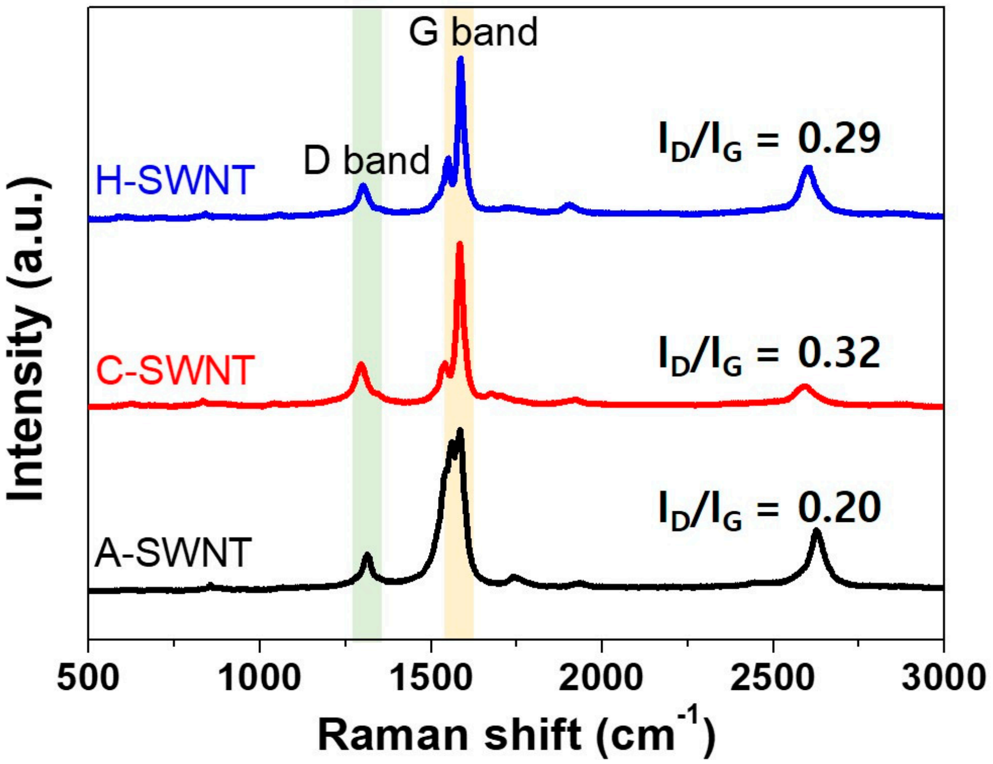

Fig. S2. Comparison of Raman spectra for different types of SWNTs (H-SWNTs, C-SWNTs, and A-SWNTs).

(a) ZnO NP/A-SWNT solution

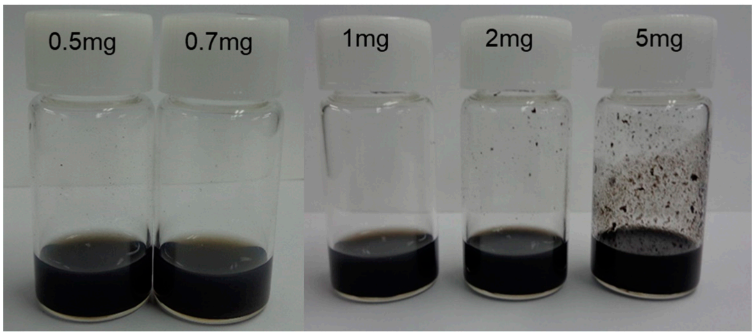

(b) ZnO NP/H-SWNT solution

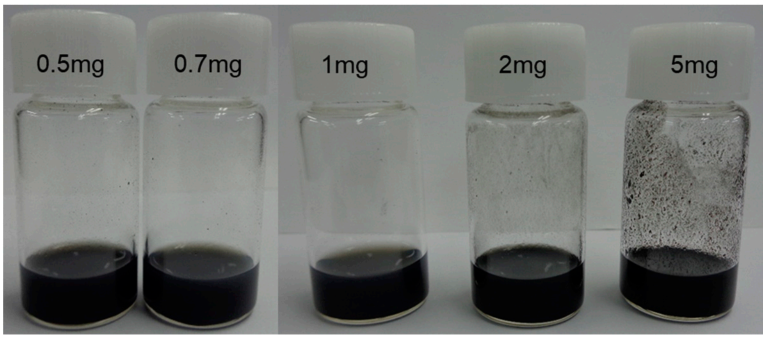

(c) ZnO NP/C-SWNT solution

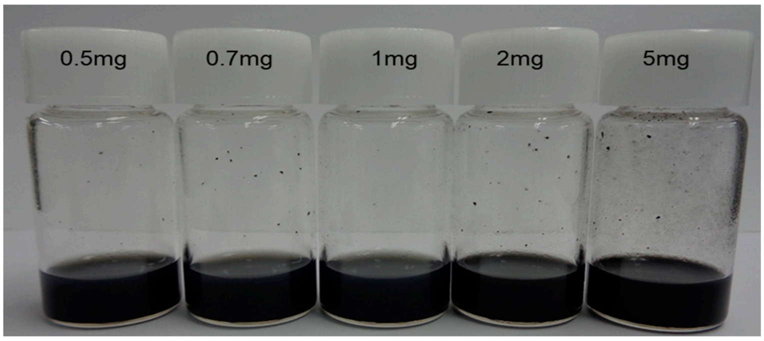

Fig. S3. Photographs of ZnO NP/SWNT solutions with different types of SWNTs (A-SWNTs, H-SWNTs, and C-SWNTs). The weights of SWNTs dispersed in 10 mg/ml ZnO NP solution (solvent: methanol + butanol) were 0.5 mg, 0.7 mg, 1.0 mg, 2.0 mg, and 5.0 mg, corresponding to the weight ratios of 40:1, 28:1, 20:1, 10:1, and 4:1 between ZnO and CNTs.

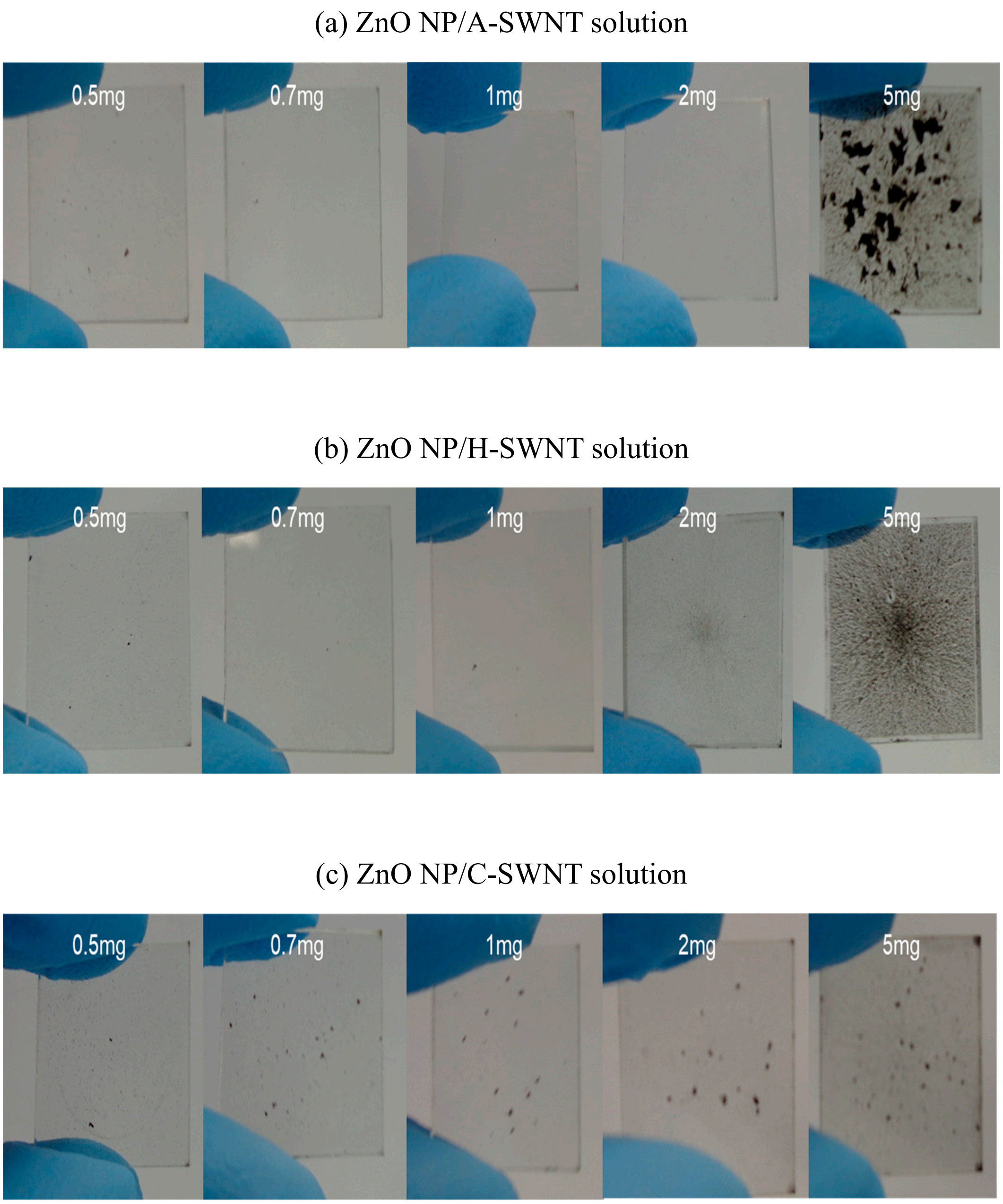

Fig. S4. Photographs of ZnO@SWNT hybrid films (films were prepared using the solution shown in Fig. S4). Therefore, the weight ratios between ZnO and CNTs were the same as those in Fig. S4 (40:1, 28:1, 20:1, 10:1, and 4:1 for 0.5 mg, 0.7 mg, 1.0 mg, 2.0 mg, and 5.0 mg SWNTs in 10 mg/ml ZnO NP solution).
